# Association of ATP-Binding Cassette Transporter A1 (*ABCA1*)-565 C/T Gene Polymorphism with Hypoalphalipoproteinemia and Serum Lipids, IL-6 and CRP Levels

**Published:** 2017

**Authors:** Mohammad Mahdi Babashamsi, Sohrab Halalkhor, Hamid Moradi Firouzjah, Hadi Parsian, Seyed Farzad Jalali, Mohammad Babashamsi

**Affiliations:** 1. Department of Biochemistry, Faculty of Medicine, Babol University of Medical Sciences, Babol, Iran; 2. Department of Molecular and Cell biology, Faculty of Basic Sciences, University of Mazandaran, Babolsar, Iran; 3. Department of Cardiology, Faculty of Medicine, Babol University of Medical Sciences, Babol, Iran; 4. Monoclonal Antibody Research Center, Avicenna Research Institute, ACECR, Tehran, Iran

**Keywords:** -565 C/T polymorphism, *ABCA1*, CRP, Hypoalphalipoproteinemia; IL-6; Lipids

## Abstract

**Background:**

ATP-binding cassette transporter A1 (*ABCA1*) is a membrane integral protein which plays a vital role in High Density Lipoprotein (HDL) metabolism and exerts a protective effect against Hypoalphalipoproteinemia (HA) by mediation of rate-limiting step in HDL biogenesis. In addition, this protein possesses anti-inflammatory effects by inhibiting the production of some inflammatory cytokines in macrophages. This study investigated the association of *ABCA1*-565 C/T gene polymorphism with HA and serum lipids, IL-6 and CRP levels.

**Methods:**

A population which consisted of 101 HA and 95 normal subjects were genotyped for *ABCA1*-565C/T polymorphism by Polymerase Chain Reaction-Restriction Fragment Length Polymorphism (PCR-RFLP). The serum concentrations of lipids, IL-6 and high sensitive-CRP (hs-CRP) were measured by the relevant methods.

**Results:**

The frequency of T allele was significantly higher in the HA group than the controls (31.7 *vs*. 19.5%, p=0.002). Thus, carriers of the T allele (CT and TT genotypes) had a higher risk for HA (p=0.016, OR=2.04, 95% CI=1.14–3.63). T allele carriers demonstrated decreased HDL-C and increased triglyceride, IL-6 and CRP levels than those with the CC genotype.

**Conclusion:**

This study suggests that the-565 C/T polymorphism of *ABCA1* gene is associated with an increased risk of HA, decreased HDL-C and increased TG, IL-6 and CRP.

## Introduction

Hypoalphalipoproteinemia (HA) is a dyslipidemia which is characterized by HDL-C concentration below 1.04 *mmol/L* (40 *mg/dL*) 1. High Density Lipoprotein (HDL) is a multifunctional lipoprotein particle which plays a vital role in the metabolism of lipids and protection against a number of diseases 2,3 by I) removing excess cholesterol from tissues and transporting them to liver for elimination; II) protecting the endothelial cell function; III) inhibiting the LDL oxidation and platelet aggregation and IV) participating in lipid metabolism by transferring the lipase cofactor and receptor. HA is associated with an increased risk of Cardiovascular Disease (CVD), type 2 diabetes mellitus, dementia, lung cancer and lymphoma 4,5. Its occurrence may be due to environmental factors and lifestyle (*e.g*. smoking, medications, overweight, physical inactivity) as well as genetic defects 6.

The most severe form of HA is the Tangier Disease (TD), a disorder which is characterized by near zero level of HDL. Homozygosity for mutations in *ABCA1* gene leads to TD 3. *ABCA1* gene is located in the q31 region of chromosome 9 and consists of 50 exons that encode a 2261 amino acid integral membrane protein 7. *ABCA1* mediates the rate-limiting step in HDL biogenesis by transporting cellular excess free cholesterol and phospholipids to an apolipoprotein acceptor 8. It is broadly expressed in macrophages, hepatocytes, intestinal cells, lung cells, adrenal gland and placental trophoblast 9. This expression is highly induced by sterols, through nuclear Liver X Receptor (LXR)/Retinoid X Receptor (RXR) pathway 10.

The accumulation of sterol-rich lipoproteins, together with their oxidation in the artery wall, results in the recruitment of macrophages and the production of inflammatory molecules 11. A study by Tang *et al* indicated that the macrophage *ABCA1*, in addition to cholesterol efflux, functioned as an anti-inflammatory receptor. The interaction of apolipoprotein (apo) A-I with *ABCA1*-expressing cells stimulates autophosphorylation (activation) of Janus kinase2 (JAK2) by an *ABCA1*-dependent mechanism. Activated JAK2 has two independent effects: I) it enhances the apo A-I binding activity of *ABCA1* which is essential for lipid export; II) Phosphorylation and activation of Signal Transducer and Activator of Transcription3 (STAT3). The activated STAT3 has anti-inflammatory function in macrophages. The phosphorylated STAT3 migrates to the nucleus and regulates the transcription of proteins which repress the production of inflammatory cytokines IL-1β, IL-6, and TNF-α 12. Regarding the increase of the mentioned inflammatory cytokines, especially IL-6 and to a lesser degree TNF-α and IL-1β, the liver and aortic endothelial cells produces C-Reactive Protein (CRP) 13.

Although the TD is rare, common Single Nucleotide Polymorphisms (SNPs) in the *ABCA1* gene are present in the general population 14. Consequently, much attention has been paid to the relationship between *ABCA1* gene polymorphisms with different phenotypes, including lipid variables and clinical endpoints 15,16. One of the common SNPs is -565C/T (rs2422493), which serves as a cytosine to thymine substitution in the promoter region of the *ABCA1* gene, which affects expression, resulting in the formation of promoter low-activity (C/T, T/T) and high-activity (C/C) genotypes^17^. The previous association studies on this polymorphism were limited to various forms of CVD, as well as changes in the lipid profile. Of course, their results were inconsistent 7,18.

As HA is the most common dyslipidemia in Iranian population 19, and moreover there exists no data yet on the association of the *ABCA1* gene polymorphisms with serum inflammatory markers, the present study investigated the association of *ABCA1*-565 C/T polymorphism with HA and serum lipids, IL-6 and CRP levels in an Iranian population.

## Materials and Methods

### Subjects

This research was approved by the Ethics Committee of Babol University of Medical Sciences and the researches undertook Helsinki treaty. Participants in the study were selected among a large number of individuals who referred to the laboratories of Babol city hospitals from January 2013 to August 2014. HA subjects were selected among the individuals with HDL-C <40 *mg/dL* and the control group among the individuals with HDL-C ≥40 *mg/dL* who were accepted to participate in the study. Written, informed consent was obtained from the volunteers. Their personal data including age, residence, past medical history, drugs, cigarette and alcohol consumption were collected through an interview. Anthropometric characteristics of the included individuals were measured and the Body Mass Index (BMI) was calculated for each individual (weight in kilograms/height in meters squared). Individuals with a BMI> 30, consumers of cigarettes, alcohol and those with diabetes, rheumatic fever, tuberculosis, infectious diseases accompanied by fever, diseases of liver and thyroid, pregnants and patients treated with lipid-lowering agents, anti-inflammatory and anti-cancer drugs were excluded from the study. The remaining 343 subjects were referred to the Cardiology department of Rouhani Hospital (Babol, Iran). Eventually, 196 subjects enrolled in the study and based on HDL-C levels, were divided into two groups: HA (HDL-C<40 *mg/dL*) and normal (HDL-C≥40 *mg/dL*).

### Sample collection

3 *ml* of blood sample was taken from each subject after overnight fasting. 1.5 *ml* of blood was transferred to a tube containing Ethylene Diamine Tetra Acetic acid (EDTA) for DNA extraction and 1.5 *ml* was transferred to a tube without any anticoagulant agent to obtain serum for biochemical analysis.

### Biochemical analysis

***Lipid profile analysis:*** The serum total cholesterol, LDL-cholesterol (LDL-C) and triglycerides were measured by enzymatic colorimetric methods. HDL-C was determined after precipitation of apoB-containing lipo-proteins with dextran sulfate and magnesium. All measurements were done using Pars Azmun diagnostic kits (Pars Azmun, Iran).

***Inflammatory factors analysis:*** CRP was determined by turbidimetric immunoassay method with auto-analyzer (Hitachi 902, Japan), using hs-CRP diagnostic kit (Pars Azmun, Iran). IL-6 level was determined using enzyme-linked immunosorbent assay (ELISA) method (Pelikine Compact human IL-6 ELISA kit, CLB, Amsterdam, the Netherlands).

***Genotyping of ABCA1-565 C/T polymorphism analysis:*** Genomic DNA was extracted from peripheral blood leukocytes using salting out method 20. Genotyping was accomplished using PCR-RFLP technique. For amplification of fragment containing-565 C/T polymorphism, a pair of forward primer 5′-CTCGGGTCC TCTGAGGGACCT-3′and reverse primer 5′-CCGCA GACTCTCTAGTCCAC-3′ (CinnaGen Co, Iran) was used. PCR reaction mixture, having a final volume of 50 *μl*, contained 2 *μl* DNA, 2 *μl* (10 *μM*) of each primers, 4 *μl* (2.5 *mM*) of dNTPs, 3 *μl* (25 *mM*) of MgC1_2_, 5 *μl* of 10× buffer, 2.5 *U* of Taq DNA polymerase (Fermentas, Burlington, USA) and the remaining was DDW. The reaction mixture was denatured at 95°*C* for 2 *min*, followed by 35 cycles of denaturation at 94°*C* for 30 *s*, annealing at 60°*C* for 60 *s* and extension at 72°*C* for 60 *s*, with a final extension at 72°*C* for 10 *min*. After amplification, aliquots of the PCR products were digested with 2U AciI restriction enzyme (Thermo scientific, USA) at 37°*C* overnight. Separation of the fragments obtained after digestion was done using 12% Polyacrylamide Gel Electrophoresis (PAGE) for 3 *hr*, under constant voltage (200 *V*). The gel was stained with silver nitrate and the bands were visualized. The AciI restriction enzyme has the ability to recognize the (C/CGC) sites. After digestion of 351 *bp* fragment, three possible genotypes were distinguished: homozygous CC (148, 130 and 73 *bp*), heterozygous CT (278, 148, 130 and 73 *bp*), and homozygous TT (278 and 73 *bp*).

### Statistical analysis

Data were analyzed statistically using the SPSS 18 software (SPSS Inc., Chicago, IL, USA), and were expressed as mean±standard deviation (M±SD). hs-CRP and IL-6 concentrations were natural log transformed to improve normality. An independent T-test was used for comparison of the demographic and biochemical variables between the two groups of HA and control. Allele frequencies were calculated by the gene counting method and differences in the frequency distributions of the alleles and genotypes between HA and control groups were evaluated by Chi-square (*χ*^2^) test. Hardy-Weinberg equilibrium (HWE) for both of the cases and control groups was assessed by the *χ*^2^ test. Multivariate logistic regression was performed to analyze the relationship between -565 C/T polymorphism and HA that were adjusted for covariates (age, BMI and sex). The biochemical parameters evaluated based on genotypes using multivariate analysis of variance (ANOVA) that were adjusted for covariates as mentioned above. The p-value less than 0.05 was considered statistically significant.

## Results

Table 1 presents the demographic and biochemical data of HA and control groups. There existed a significant difference in total cholesterol, triglycerides, HDLC, LDL-C, IL-6 and hs-CRP between the two groups (p<0.05), however, there were no significant differences between the two groups in sex, age and BMI (p> 0.05).

**Table 1.** The demographic and biochemical data of the study population

	**HA group (n=101)**	**Control group (n=95)**	**p-value**
**Sex (M:F)**	52:49	49:46	0.67
**Age (years)**	44.90±11.61	47.09±10.30	0.16
**BMI (*Kg/m*^2^)**	27.68±2.06	26.85±2.43	0.240
**Total cholesterol (*mg/dL*)**	195.26±37.90	185.10±25.21	0.030 ^*^
**Triglycerides (*mg/dL*)**	163.76±53.23	142.36±48.13	0.004 ^†^
**LDL- C (*mg/dL*)**	125.29±30.49	115.87±31.99	0.036 ^*^
**IL-6 (*pg/mL*)**	1.72±2.07	1.12±1.68	0.028 ^*^
**hs- CRP (*mg/dL*)**	0.251±0.267	0.174±0.225 0.031	^*^

n: number of individuals

The results are shown as mean±SD;

* p<0.05,

† p<0.01.

The frequency distribution of the alleles and genotypes in *ABCA1*-565 C/T polymorphism between HA and control groups, are shown in table 2.

**Table 2.** Genotype and allele frequencies of the -565 C/T gene polymorphism in HA and control group

	**HA group (n=101)**	**Control group (n=95)**	p-value *
**Genotypes**			
CC n (%)	48 (47.5)	62(65.3)	
CT n (%)	42 (41.6)	29(30.5)	0.027 ^*^
TT n (%)	11 (10.9)	4(4.2)	
**Alleles**			
C allele n (%)	138 (68.3)	153 (80.5)	0.002^†^
T allele n (%)	64 (31.7)	37 (19.5)	

n: number of individuals;

* p<0.05,

† p<0.01

a: *χ*^2^-test for distributions of genotype and allele frequencies between the HA and control group.

There was consistency in the frequency distribution of -565 C/T polymorphism in the HA and control groups with the HWE. It also showed that the frequency of T allele was significantly higher among the HA group in comparison with the controls. In addition, the results illustrated that the distribution of genotypes between the two groups was significantly different.

A multivariate logistic regression was performed to evaluate the association of -565 C/T polymorphism with HA. When the CC genotype was used as a reference, the TT and CT genotypes were both significantly associated with HA risk. So that, a significant association was observed for T allele carrier genotypes when compared with CC genotype for HA risk (Table 3).

**Table 3.** Association of *ABCA1*-565 C/T polymorphism with HA

	**OR ^a^**	**95% CI**	**p-value**
**CC**		(ref)	
**CT**	1.84	1.01–3.38	0.046^*^
**TT**	3.44	1.02–11.49	0.044^*^
**CT+TT**	2.04	1.14–3.63	0.016^*^

a: Adjusted ORs were obtained from a multivariate logistic regression with adjustment for age, sex and BMI.

* p<0.05.

The analysis of biochemical parameters, based on genotypes, demonstrated that the T allele carriers exhibited significantly lower HDL-C levels and significantly higher TG, IL-6 and hs-CRP levels than those with the CC genotype (p<0.05), while the total cholesterol and LDL-C were not significantly different among genotypes (p>0.05, Table 4).

**Table 4.** The levels of biochemical parameters according to the genotype

	**CT and TT (n=86)**	**CC (n=110)**	**p-value**
**Total cholesterol (*mg/dL*)**	193.35±33.1	187.05±29.57	0.134
**Triglycerides (*mg/dL*)**	159.37±51.62	146.78±47.94	0.041 ^*^
**HDL- C (*mg/dL*)**	39.65±4.81	41.33±5.21	0.018 ^*^
**LDL- C (*mg/dL*)**	124.48±29.23	117.29±32.2	0.085
**IL-6 (*pg/mL*)**	1.58±1.92	1.14±1.68	0.047 ^*^
**hs-CRP (*mg/dL*)**	0.250±0.275	0.176±0.236	0.048 ^*^

The differences between means were analyzed by multivariate ANOVA adjusted for age, sex and BMI.

* p<0.05.

## Discussion

The results of the current study showed an association between -565 C/T polymorphism and the risk of HA. Based on the results, individuals carrying the T allele were more susceptible to HA.

Previous studies investigated the effect of -565 C/T polymorphism on HA-related diseases, such as various types of CVD. The results of these studies have been controversial. Lutacuta *et al* showed that the presence of the T allele in -565 C/T locus was associated with the severity of the atherosclerosis in patients with established Coronary Artery Disease (CAD) 18. Benton *et al* in a Multi-Ethnic Study of Atherosclerosis (MESA) reported a borderline association between the presence of T allele and higher prevalence of the coronary artery calcification 14. In contrast, Jensen *et al* reported an inverse relationship between the -565 C/T variant with the risk of Coronary Heart Disease (CHD) among healthy women 21.

Of course, there existed no association in a number of studies. For example, in a study of 316 Tunisian patients who underwent coronary angiography, according to Rejeb *et al* there was no significant association between -565 C/T polymorphism with the stenosis risk 7. In addition, Takagi *et al* found no relationship between -565 C/T with myocardial infarction or angina pectoris in Japanese patients 22.

The reason for these conflicting results obtained in various studies is still unclear. Racial difference of different populations might be one of the major reasons for disputes. In addition, the presence or absence of an observed association in any race, ethnicity or geographic populations may be related to a number of other factors, such as gene-gene interactions and environmental factors.

Also, there have been contradictory results regarding the association between -565 C/T variant and lipid profile. While some investigators did not find any significant association between the polymorphism and lipid profile 7,14,18, Liu *et al* found a significant decrease of HDL-C concentrations in TT genotype compared to those with the CC genotype 23. In the present study, the assessment of lipid profile based on genotype showed that T allele carriers had significantly lower HDL-C and higher TG levels than those with the CC genotype. In addition, this study evaluated the association of -565 C/T polymorphism with IL-6 and CRP levels for the first time. The results revealed the association between this polymorphism, with a significant increase of IL-6 and CRP levels. The -565 C/T polymorphism is located in the promoter region. Hence, a possible mechanism for the observed associations may be due to the effect of -565 C/T polymorphism on the *ABCA1* gene expression level. Studies of Kyriakou *et al* on the *ABCA1* expression levels in *ex vivo* atherosclerotic tissue of individuals with different genotypes of -565 C/T exhibited a lower expression level in T allele carriers. In addition, *in vitro* studies in macrophages revealed remarkable results. Transient transfection and reporter assays demonstrated a lower activity of the T allelic promoter in driving gene expression than the C allelic promoter. Also, investigation of allele specific effect of the -565 C/T polymorphism on binding of nuclear proteins to the *ABCA1* gene promoter indicated that this variant was located in a transcription factor binding site and lower binding at the presence of the T allele 17. These data suggest that the polymorphism -565 C/T may have effect on the expression of *ABCA1* and result in a reduction in the transportation of cholesterol and anti-inflammatory activity; resulting in decreased HDLC and increased IL-6, and consequently CRP.

The increase of TG in T allele carriers of the -565 C/T variant is remarkable. The activity of Cholesterol Ester Transfer Protein (CETP) causes the equilibration of the core components of lipoprotein particles. Cholesteryl esters are transferred from HDL to TG-rich lipoproteins, while TG is transferred by TG-rich lipoproteins to HDL. Decreased *ABCA1* activity, resulting in decreased HDL-C, may decrease the cholesteryl ester/TG exchange. TG component of HDL is hydrolyzed by hepatic lipase. Thus, decreased transfer of TG to HDL may ultimately decrease the TG catabolism 24. In addition, studies on *ABCA1* deficient mice suggested the role of hepatocyte *ABCA1* in TG metabolism. Based on these results, large nascent HDL particles assembled by hepatocyte *ABCA1* bind to a putative membrane receptor (Topβ, target of pre-β), which in turn stimulates PI3 kinase activation and as a result reduces the lipid mobilization and secretion of normalsized VLDL particles (VLDL2). In the absence of hepatic *ABCA1* or diminished *ABCA1* activity, reduced nascent HDL particle formation leads to diminished signaling through Topβ, which results in a reduction of PI3 kinase activation, increased lipid mobilization, and increased secretion of larger TG-enriched VLDL particles (VLDL1) 25,26. Thus, it is possible that the impact of -565 C/T polymorphism on HDL-C levels results to an increase in the TG level.

## Conclusion

In conclusion, this study suggests that the *ABCA1*-565 C/T gene polymorphism is associated with an increased risk of HA, decreased HDL-C and increased TG, IL-6and CRP. Due to the association of HA with a number of diseases, the identification of genetic risk factors associated with HA is an opportunity to identify individuals that are susceptible to this disorder, and also for pre-emptive measures to be taken in order to prevent HA, and consequently the related diseases. On the other hand, an evaluation of the *ABCA1* variants association with the inflammatory factors can lead to the recognition of the agent involved in progression of inflammation. Hence, further study with larger sample sizes is recommended in order to confirm the findings of this study.

## References

[1] Expert Panel on Detection, Evaluation, and Treatment of High Blood Cholesterol in Adults Executive summary of the third report of the National Cholesterol Education Program (NCEP) expert panel on detection, evaluation, and treatment of high blood cholesterol in adults (Adult Treatment Panel III). JAMA 2001; 285 (19): 2486– 2497. 11368702 10.1001/jama.285.19.2486

[2] Liu Y Tang C. Regulation of ABCA1 functions by signaling pathways. Biochim Biophys Acta 2012; 1821 (3): 522– 529. 21920460 10.1016/j.bbalip.2011.08.015 PMC3243790

[3] Oram JF. Molecular basis of cholesterol homeostasis: lessons from Tangier disease and ABCA1. Trends Mol Med 2002; 8 (4): 168– 173. 11927274 10.1016/s1471-4914(02)02289-x

[4] Alharbi KK Khan IA Al-Daghri NM Munshi A Sharma V Mohammed AK ABCA1 C69T gene polymorphism and risk of type 2 diabetes mellitus in a Saudi population. J Biosciences 2013; 38 (5): 893– 897. 10.1007/s12038-013-9384-x 24296892

[5] McGrowder D Riley C Morrison EYSA Gordon L. The role of high-density lipoproteins in reducing the risk of vascular diseases, neurogenerative disorders, and cancer. Cholesterol 2010; 2011: 496925. 21490772 10.1155/2011/496925 PMC3065895

[6] Pisciotta L Hamilton-Craig I Tarugi P Bellocchio A Fasano T Alessandrini P Familial HDL deficiency due to ABCA1 gene mutations with or without other genetic lipoprotein disorders. Atherosclerosis 2004; 172 (2): 309– 320. 15019541 10.1016/j.atherosclerosis.2003.11.009

[7] Rejeb J Omezzine A Rebhi L Boumaiza I Kchock K Belkahla R Associations between common polymorphisms of adenosine triphosphate-binding cassette transporter A1 and coronary artery disease in a Tunisian population. Arch Cardiovascular Diseases 2010; 103 (10): 530– 537. 10.1016/j.acvd.2010.10.003 21130966

[8] Brunham LR Kruit JK Pape TD Timmins JM Reuwer AQ Vasanji Z β-cell ABCA1 influences insulin secretion, glucose homeostasis and response to thiazolidinedione treatment. Nat Med 2007; 13 (3): 340– 347. 17322896 10.1038/nm1546

[9] Yin K Liao DF Tang CK. ATP-binding membrane cassette transporter A1 (ABCA1): a possible link between inflammation and reverse cholesterol transport. Mol Med 2010; 16 (9–10): 438. 20485864 10.2119/molmed.2010.00004 PMC2935947

[10] Oram JF Lawn RM. ABCA1: the gatekeeper for eliminating excess tissue cholesterol. J Lipid Res 2001; 42 (8): 1173– 1179. 11483617

[11] Hansson GK Robertson A-KL Söderberg-Nauclér C. Inflammation and atherosclerosis. Annu Rev Pathol 2006; 1: 297– 329. 18039117 10.1146/annurev.pathol.1.110304.100100

[12] Tang C Liu Y Kessler PS Vaughan AM Oram JF. The macrophage cholesterol exporter ABCA1 functions as an anti-inflammatory receptor. J Biol Chem 2009; 284 (47): 32336– 3243. 19783654 10.1074/jbc.M109.047472 PMC2781648

[13] Francisco G Hernández C Simó R. Serum markers of vascular inflammation in dyslipemia. Clin Chim Acta 2006; 369 (1): 1– 16. 16469304 10.1016/j.cca.2005.12.027

[14] Benton JL Ding J Tsai MY Shea S Rotter JI Burke GL Associations between two common polymorphisms in the ABCA1 gene and subclinical atherosclerosis: Multi-Ethnic Study of Atherosclerosis (MESA). Atherosclerosis 2007; 193 (2): 352– 360. 16879828 10.1016/j.atherosclerosis.2006.06.024

[15] Castaño-Jameson M Guevara M Flores A Medina I Ayala B Aguilar M Association between ABCA1 R230C polymorphism and biochemical parameters in subjects with metabolic syndrome (641.7). The FASEB J 2014; 28 (1 Supplement): 641– 647.

[16] Daimon M Kido T Baba M Oizumi T Jimbu Y Kameda W Association of the ABCA1 gene polymorphisms with type 2 DM in a Japanese population. Biochem Biophys Res Commun 2005; 329 (1): 205– 210. 15721294 10.1016/j.bbrc.2005.01.119

[17] Kyriakou T Hodgkinson C Pontefract DE Iyengar S Howell WM Wong YK Genotypic effect of the -565C> T polymorphism in the ABCA1 gene promoter on ABCA1 expression and severity of atherosclerosis. Arterioscler Thromb Vasc Biol 2005; 25 (2): 418– 423. 15528481 10.1161/01.ATV.0000149379.72018.20

[18] Lutucuta S Ballantyne CM Elghannam H Gotto AM Jr Marian AJ. Novel polymorphisms in promoter region of ATP binding cassette transporter gene and plasma lipids, severity, progression, and regression of coronary atherosclerosis and response to therapy. Circ Res 2001; 88 (9): 969– 973. 11349008 10.1161/hh0901.090301

[19] Halalkhor S Mesbah-Namin SA Daneshpour MS Hedayati M Azizi F. Association of ATP-binding cas-sette transporter-A1 polymorphism with apolipoprotein AI level in Tehranian population. J Genetics 2011; 90 (1): 129. 21677398 10.1007/s12041-011-0030-9

[20] Miller SA Dykes DD Polesky HF. A simple salting out procedure for extracting DNA from human nucleated cells. Nucleic Acids Res 1988; 16 (3): 1215. 3344216 10.1093/nar/16.3.1215 PMC334765

[21] Jensen MK Pai JK Mukamal KJ Overvad K Rimm EB. Common genetic variation in the ATP-binding cassette transporter A1, plasma lipids, and risk of coronary heart disease. Atherosclerosis 2007; 195 (1): e172– e180. 17368464 10.1016/j.atherosclerosis.2007.01.025

[22] Takagi S Iwai N Miyazaki S Nonogi H Goto Y. Relationship between ABCA1 genetic variation and HDL cholesterol level in subjects with ischemic heart diseases in Japanese. Thromb Haemost 2002; 88 (2): 369– 370. 12195719

[23] Liu S Guo Z Lai W Tu Y Chen J. [Distribution of-477C/T single nucleotide polymorphism in the promoter region of ABCA1 gene and its significance for plasma lipids levels in normal Chinese Han population]. Di 1 jun yi da xue xue bao= Academic journal of the first medical college of PLA 2004; 24 (6): 650– 652. 15201080

[24] Tall AR Yvan-Charvet L. Cholesterol, inflammation and innate immunity. Nat Rev Immunol 2015; 15 (2): 104– 116. 25614320 10.1038/nri3793 PMC4669071

[25] Liu M Chung S Shelness GS Parks JS. Hepatic ABCA1 and VLDL triglyceride production. Biochim Biophys Acta 2012; 1821 (5): 770– 777. 22001232 10.1016/j.bbalip.2011.09.020 PMC3272310

[26] Chung S Gebre AK Seo J Shelness GS Parks JS. A novel role for ABCA1-generated large pre-β migrating nascent HDL in the regulation of hepatic VLDL triglyceride secretion. J Lipid Res 2010; 51 (4): 729– 742. 20215580 10.1194/jlr.M900083 PMC2842152

